# Modelling non-Markovian dynamics in biochemical reactions

**DOI:** 10.1186/1752-0509-9-S3-S8

**Published:** 2015-06-01

**Authors:** Davide Chiarugi, Moreno Falaschi, Diana Hermith, Carlos Olarte, Luca Torella

**Affiliations:** 1 Dipartimento di Ingegneria dell'Informazione e Science Matematiche, Università degli Studi di Siena, Via Roma 56, 53100 Siena, Italy; 2 Max Planck Institute of Colloids and Interfaces, Am Muhlenberg 1, 14476 Potsdam, Germany; 3 ECT, Universidade Federal do Rio Grande do Norte, Campus Universitario Av. Senador Salgado Filho, Natal, Brazil; 4 Pontificia Universidad Javeriana Cali, Calle 18 No 118-250, Cali, Colombia

**Keywords:** Non-Markovian dynamics, constraint programming, biochemical reactions

## Abstract

**Background:**

Biochemical reactions are often modelled as discrete-state continuous-time stochastic processes evolving as memoryless Markov processes. However, in some cases, biochemical systems exhibit non-Markovian dynamics. We propose here a methodology for building stochastic simulation algorithms which model more precisely non-Markovian processes in some specific situations. Our methodology is based on Constraint Programming and is implemented by using Gecode, a state-of-the-art framework for constraint solving.

**Results:**

Our technique allows us to randomly sample waiting times from probability density functions that not necessarily are distributed according to a negative exponential function. In this context, we discuss an important case-study in which the probability density function is inferred from single-molecule experiments that describe the distribution of the time intervals between two consecutive enzymatically catalysed reactions. Noticeably, this feature allows some types of enzyme reactions to be modelled as non-Markovian processes.

**Conclusions:**

We show that our methodology makes it possible to obtain accurate models of enzymatic reactions that, in specific cases, fit experimental data better than the corresponding Markovian models.

## Background

Experimental evidence at the single-cell level suggest that random fluctuations at the microscopic scale can have an important impact in determining the complex behaviour of living organisms [1,2]. These findings have raised significant interest towards discrete stochastic (DS) models of biological systems as a tool for bridging the gap between stochastic events arising at the molecular level and the corresponding macroscopic phenomena. In DS models, the system evolves according to a stochastic algorithm which samples the probability of the next state transition from a given probability density function (PDF). Biochemical reactions, in particular, are often modelled as discrete-state, continuous-time Markov Processes (CTMP). This method represents an alternative to the traditional continuous deterministic modelling (CDM) approach when random fluctuations must be properly taken into account. This is the case, for instance, of systems composed of a small number of elements like molecular subsystems in living cells (e.g., metabolic networks, signalling pathways, or gene regulatory networks). In this context, descriptions provided by reaction rate equations fail both to predict the fluctuations in molecular populations and to capture stochastic-driven phenomena such as Stochastic Focusing [3], Stochastic Switching [4] and Multiplicative Noise Effects [5]. The Gillespie's Stochastic Simulation Algorithm (GSSA) [6,7], based on [8] and [9], is probably the most popular algorithm used for simulating DS models of (bio)chemical systems. The GSSA relies on a Monte Carlo technique, namely the Inverse Transform Sampling (ITS). GSSA is used to numerically simulate the Markov process described analytically by the set of differential equations from the Chemical Master Equation (CME). Since the CME can rarely be solved either numerically or analytically especially for large systems, GSSA provides a computational method to generate statistically correct trajectories (possible solutions) of the CME. These trajectories are obtained by drawing random samples from the so-called Reaction Probability Density Function (RPDF) [6,7] through the ITS.

Gillespie's SSA is designed for simulating sets of *elementary *chemical reactions occurring in a well-stirred mixture in a fixed volume and at a constant temperature [10]. Thus, the straightforward application of GSSA to a biochemical context can be difficult. Indeed, it is easy to realise the difficulties in specifying all the elementary reactions composing a given biochemical system, mainly because of the lack of needed information. Indeed, only macroscopic and mesoscopic events can be observed experimentally and, thus, it is not possible to know the complete list of elementary reactions. The usual strategy for tackling this problem consists of abstracting away the non-observable elementary steps and replacing them with a single reaction event rendered as a "Markov jump" with the transition time (*τ *) sampled from a negative exponential distribution. Even though heuristic, this strategy can lead to simulation results showing a good agreement with experimental data (see e.g., [11-13]).

However, the impact of the approximations introduced by this abstraction process is difficult to evaluate or estimate, as reported by Gillespie in [13] for enzymatically catalysed reactions. One crucial issue of this abstraction-approximation strategy is related to the modelling of the time needed for a reaction to occur: even though each of the elementary reactions composing a biochemical system can be described as a CTMP (and, thus, with transition times probabilities following a negative exponential PDF), the waiting times between two subsequent events observed at a mesoscopic or macroscopic scale can be non-exponential, as reported, e.g., in [14,15] and confirmed by experimental evidence [16,17]. It can be demonstrated (see e.g., [18] that a stochastic process exhibiting nonexponential waiting times does not enjoy necessarily the so-called *Markov Property *(a.k.a. *memoryless property*) and thus, strictly speaking, it cannot be a Markov process. These arguments suggest the need for modelling frameworks that allow us to deal with a more general notion of waiting times, thus managing non-memoryless (non-Markovian) systems' evolutions. In terms of waiting times, this corresponds to considering frameworks in which the PDF describing transition times can be different from a negative exponential.

Various proposals have been developed for addressing the aforementioned issues. BioPEPAd [15] offers the possibility of adding deterministic delays to the duration of a reaction. In [19] an extension for process calculi is proposed, allowing the expression of activity durations through general probability distributions. A similar approach is proposed in [20] for extending Petri Nets. Furthermore, in [14] the authors improved the Beta Workbench (BWB) framework with the possibility of sampling waiting times from PDFs that are different from the negative exponential, such as the Erlang or Hyperexponential. This strategy results in better matches with the observed non-Markovian biological behaviours. However, even though the data-fitting can enhance results, the considered PDFs do not exactly match the experimental evidence. Hence, they may themselves introduce unpredictable approximations.

In this paper, we propose a Constraint Programming approach that is suited for being embedded in Monte Carlo algorithms for discrete-state continuous-time stochastic simulation of biochemical reactions. Our method allows us to sample random numbers from PDFs that may not necessarily follow a negative exponential distribution including, e.g. the Erlang and Hyperexponential distributions considered in [14].

Relying also on the results about single-molecule enzyme reactions presented in [21], we exploit our method for efficiently and accurately simulating the occurrence of enzyme catalysed reactions that follows the Michaelis-Menten (MM) scheme. Noticeably, our approach allows us to simulate this kind of reactions as a single *S*→ *P *(Substrate *→ *Product) step without any loss of accuracy, i.e., without introducing approximations.

The contribution of this paper is hence twofold: on the one hand we propose a general method for building discrete-state continuous-time stochastic simulations of (bio)chemical systems, sampling waiting times from general PDFs distributions; on the other hand, exploiting our method, we provide an efficient and accurate strategy for the stochastic simulation of MM enzyme reactions. In the latter case, we provide a simulation technique that overcomes some of the limitations of the corresponding GSSA approach.

Our method is implemented on top of the Gecode library (http://www.gecode.org), an efficient framework to solve constraint satisfaction problems [22,23]. Essentially, we use the real intervals constraint support of Gecode for solving sets of interval constraints. Although specific numerical methods may be computationally more efficient, declarative methods as Constraint Programming provide a flexible framework able to quickly adapt to different situations, as we explain later. Hence, the modellers have at their disposal a more general (software) tool that minimises the need for writing new code when the parameters of the model change.

We will show the features of our method through its application tot the simulation of enzyme reactions following the MM scheme. To do this, we first introduce in the following subsection some details regarding the MM reaction scheme and the results about single-molecule enzyme reactions obtained in [21].

### Simulating Single-Molecule Enzyme Reactions

A convenient (and popular) way of describing all the elementary steps of an enzymatically catalysed reaction is through the Michaelis-Menten (MM) model. According to this reaction scheme, a catalysed reaction of the kind *S*→ *P *(where *S *and *P *are the substrate and product molecules respectively, while *E *represents the enzyme molecule and *ES *the enzyme-substrate complex) can be approximated with three "elementary" reactions:

(1) $$E+S\overset{k_{1}}{\underset{k_{-1}}{⇌}}ES\overset{k_{2}}{\to }E+P$$

The reaction set (1) can be simulated as a CTMP via GSSA using *k*_1_, *k*_*−*1 _and *k*_2 _as "elementary" rate constants. It is described as a single Markov step and, thus, the corresponding transition time follows a negative exponential PDF. The propensity is calculated through Equation (2), where *v *is the rate of product formation, [*S*] the substrate concentration and *K_M _*the MM constant (see, e.g., [13]).

$$v=\frac{k_{\text{2}}\left[S\right]}{\left[S\right]+K_{M}}$$

MM kinetics is particularly convenient because it effectively reduces the three reactions in Equation (1) to a single reaction *S *→ *P *with rate *v*. Moreover, the necessary parameters are easier to measure experimentally than the rate constants *ki*.

However, modelling enzymatic reactions with the MM kinetics introduces approximations at two different levels. The first level of approximation is inherent to the MM kinetics itself. Indeed, it should be noted that Equation (2) does not capture the dynamics of reaction set (1) exactly, being based on assumptions (e.g., the steady-state assumption [24]) that are approximately valid. The steady-state approximation is the first important assumption involved in the MM reaction scheme. According to this approximation, the concentration of the *ES *complex will rapidly reach the steady-state, i.e., after an initial burst phase, the concentration of *ES *will not change appreciably until a significant amount of substrate is consumed [25]. In *in vivo *experiments this assumption may not necessarily hold because the enzyme concentrations can be comparable to the substrate concentrations [26]. The steadystate approximation is well studied in the deterministic setting, where the reaction set (1) is described through a set of ordinary differential equations (ODEs).

Building a stochastic model *à la *Gillespie of the reaction scheme (1), relying upon MM kinetics, requires the conversion of an ODE model into a stochastic model. As previously pointed out, this is straightforward if the ODEs describe elementary reactions. Some authors (see e.g., [12]) compared the output of a stochastic model that uses MM kinetics and the corresponding model decomposed into the three "elementary" reactions in (1), and no significant differences in simulation results were found. The work in [11] verified the equivalence of the deterministic and the stochastic MM approximations under a restricted set of initial conditions. In spite of these results, however, there is no general (theoretical) method for converting MM terms from the deterministic to the stochastic setting. This problem has been exhaustively studied in [13], where it is shown that a "full" stochastic model of the reaction set in Equation (1), describing explicitly the three reactions, can be safely reduced to one *S*→ *P *reaction with stochastic rate *v *under certain conditions, namely, after the pre-steady-state transient period.

The second level of approximation regards the Markovian modelling paradigm. Modelling the reaction set in Equation (1) as a single Markov jump defined by the lumped reaction *S*→ *P *with rate *v*, implies subsuming that the process satisfies the Markov (or *memoryless*) property: the probability of transition from the current state to one of the possible successors depends only on the current state. This, in turn, means that the PDF describing the waiting time *τ *must be a negative exponential function. As showed by single-molecule experiments [17,21], the PDF describing the occurrence of *τ *is significantly different from an exponential. In other words, experimental evidence suggests that the occurrence of an enzymatically catalysed reaction is not a Markov process. The problem of evaluating the approximation introduced by Markovian models of natural systems is well known in physics and in some cases can be quantified [27].

In the cases that have been studied, the waiting time *τ *for a biochemical reaction catalysed by a single enzyme results to be distributed according to the following PDF:

(3) $$f\left(\tau \right)=\frac{k_{\text{1}}k_{\text{2}}\left[S\right]}{\text{2}A}\left[e^{\left(A+B\right)\tau }-e^{\left(B-A\right)\tau }\right]$$

where [S] is the substrate concentration, *k*_1_, *k*_*−*1_, *k*_2 _and are the kinetic constants (see Equation (1)) and

$$A=\sqrt{\frac{{\left(k_{\text{1}}\left[S\right]+k_{-1}+k_{\text{2}}\right)}^{\text{2}}}{4}-k_{\text{1}}k_{\text{2}}\left[S\right]}\;\;B=\frac{-\left(k_{\text{1}}\left[S\right]+k_{-1}+k_{\text{2}}\right)}{2}$$

We note that A>0 and B<0. Differently from the classical ensemble experiments (in which the variations of reactive species concentrations are measured in solutions containing "large" amounts of molecules), a single-molecule experiment records the stochastic time trace of repetitive reactions of an individual enzyme molecule. In particular (see [21] and [17] for details) the measured quantities regard the duration of the *waiting time *that passes between a reaction and the following one. Therefore, in the absence of dynamic disorder, *f *(*τ *) describes the temporal behaviour of the single-molecule MM system in Equation (3) at any specified substrate concentration. This PDF is exact and does not invoke the steady-state assumption, but it can be reduced to the steady-state case [17].

Note that Equation (3) is very different from the negative exponential PDF for waiting times proposed by Gillespie. This suggests that GSSA may be not adequate to simulate biochemical reactions catalysed by enzymes. Another remarkable fact to notice is that the reciprocal of the mean waiting time $\left(\frac{1}{\left\langle \tau \right\rangle }\right)$obeys a MM-type equation (called the *Single-Molecule-Michaelis-Menten-Equation*):

(4) $$\frac{1}{\left\langle \tau \right\rangle }=-\frac{{\left(A^{\text{2}}-B^{\text{2}}\right)}^{\text{2}}}{\text{2}Bk_{\text{1}}k_{\text{2}}\left[S\right]}=\frac{k_{\text{2}}\left[S\right]}{\left[S\right]+K_{M}}$$

**Figure 1:**
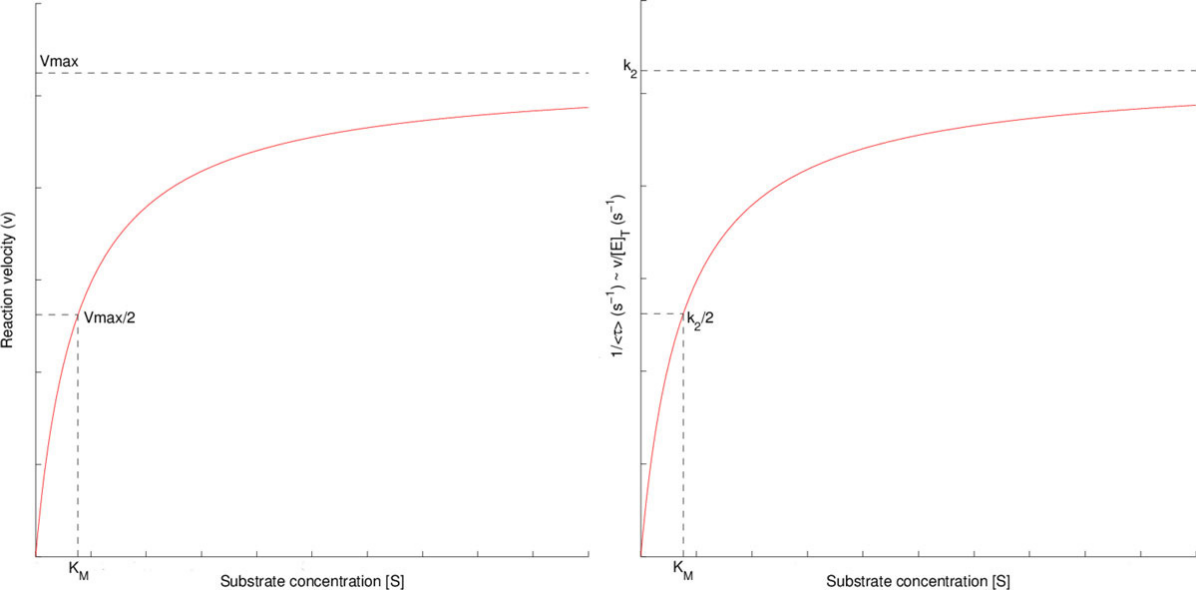
**Plots of [*S*] vs**. *v *and [*S*] vs. $\frac{1}{\left\langle \tau \right\rangle }$ obeying a MM-type equation (adapted from [17,28]).

Figure 1 (right) shows a plot of [*S*] vs $\frac{1}{\left\langle \tau \right\rangle }$. Note that this plot exhibits the characteristic hyperbolic profile of the classical MM saturation curve. The reader may wish to compare it with Figure 1 on the left, which is a plot of [*S*] vs *v *drawn from the Equation (2), that refers to the MM model for enzymatically catalysed reactions in Equation (1). The fact that the fraction $\frac{1}{\left\langle \tau \right\rangle }$calculated from Equation (4) exactly coincides with Equation (2) highlights the ergodicity of the process, i.e., the consistency between the single-molecule and the ensemble-averaged kinetics. This issue is also evident by the fact that the same constants (*k*_1_, *k*_*−*1_, *k*_2 _and *K_M _*) appear both in Equation (2) and Equation (4). This correspondence is useful for computer-based stochastic simulations of single-molecule reactions because it allows the safe use of the constants *k*_1_, *k*_*−*1_, *k*_2 _and *K_M _*measured in the "ensemble" experiments.

In the next section, we show how our technique allows us to build a Monte Carlo-based algorithm for simulating the occurrence of (networks of) single-molecule enzymatic reactions, by sampling waiting times from Equation (3) through the ITS method.

## Methods

### Sampling waiting times through the ITS method

For the sake of readability, let us write the Equation (3) as:

(5) $$f\left(\tau \right)=\alpha \;\left[e^{\left(\beta \tau \right)}-e^{\left(\gamma \tau \right)}\right]$$

where β≥γ and

$$\alpha =\frac{k_{\text{1}}k_{\text{2}}\left[S\right]}{\text{2}A}\;\;\beta =\left(A+B\right)\;\;\gamma =\left(B-A\right)$$

Let $F\left(\tau \right)\in \left[0..\text{1}\right]$ be the cumulative distribution function for $f\left(\tau \right)$:

(6) $$F\left(\tau \right)=\int_{-\infty }^{\tau }f\left(\tau \right)d\tau =\alpha \left[\frac{e^{\left(\beta \tau \right)}}{\beta }-\frac{e^{\left(\gamma \tau \right)}}{\gamma }\right]-\alpha \left[\frac{\text{1}}{\beta }-\frac{\text{1}}{\gamma }\right]=r$$

At this point, the ITS technique requires to find those values of *τ *such that *F *(*τ *) = *r *by finding an analytical expression for the equation *τ *= *F *^*−*1^(*r*). Unfortunately, it is only possible to obtain such an expression in some particular cases, e.g. when *β *= 2*_γ_*.

**Figure 2:**
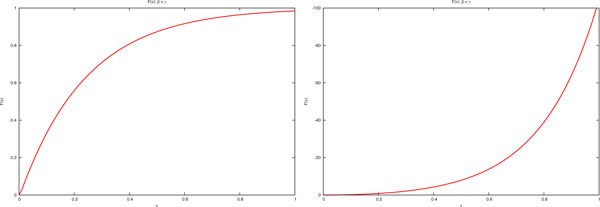
**Plots of the function *F *(*τ *) in Equation (6)**. (a): Case *β > γ*. (b): Case *β < γ*.

It is easy to see that *F *(*τ *) (*τ >*0) satisfies the following:

- for *β < γ *the plot of *F *(*τ *) is the one depicted in Figure 2 on the right and so the equation *F *(*τ *) = *r *(being 0 *< r <*1) has no solutions.

- for *β > γ *the plot of *F *(*τ *) is that depicted in Figure 2 on the left and so the equation *F *(*τ *) = *r *(being 0 *< r <*1) has certainly one (and only one) solution for each *r *when *τ >*0. This solution is rarely available analytically but it can be estimated through numerical methods.

In the next section, we present an efficient method based on Constraint Programming for computing a solution to the equation *τ *= *F *^*−*1^(*r*). This technique will be shown to be useful for sampling the waiting times taken from general PDFs. Then, we shall describe our method based on experiments of individual enzyme molecules, reporting the obtained simulation results. In particular, we will compare our results with the homologous ones presented in [13] in order to evaluate the differences between Markovian (à la Gillespie) and non-Markovian simulations in our case study.

### The Constraint Programming Approach

As we have seen, the ITS method requires to compute a solution for *F *(*τ *) = *r *given a set of values for the constants of the density function. Then, it generates a set of random values for *τ *sampled from Equation (3), simulating for *i*-times the occurrence of a single-molecule enzymatic reaction, with parameters *k*_1_, *k*_2 _and *k*_3 _(*k*_3 _is used to refer to *k*_*−*1_). To solve these equations, we use the Gecode library (http://www.gecode.org), a state-of-the-art framework for Constraint Programming including a real intervals constraint system (Float variables). Constraint systems are at the heart of Constraint Logic Programming [22,23] where problems are solved declaratively: one states the problem and a Search Engine searches *automatically *for a solution. In Constraint Programming, constraints are asserted by means of *propagators *that prune the domain of the variables through efficient techniques such as Hull, Box and *kB−*Consistency ( [29], [30]), thus allowing for solving sets of interval constraints.

An interesting feature of this programming paradigm is that constraints represent *relations *between the variables rather than *assignments *to values. The function of the propagators is then to discard the values on the domain of the variables that are not part of any solution (domain narrowing). Let us explain this situation with a simple example. Assume, for instance, three variables *x*_1_*, x*_2_*, x*_3 _with domains (intervals) [*l*_1_*, u*1], [*l*_2_*, u*_2_], [*l*_3_*, u*_3_], respectively. If we add the constraint *x*_1 _+*x*_2 _= *x*_3_, the propagators implementing the relation between the expression "*x*_1 _+ *x*_2_" and the right-hand side *x*_3 _will prune the domains of the three variables to assert that [*l*_1_*, u*_1_] + [*l*_2_*, u*_2_] = [*l*_3_*, u*_3_]. Hence, *l*_3 _*≥ l*_1 _+ *l*_2 _and *u*_3 _*≤ u*_1 _+ *u*_2_.

Rarely, propagation of constraints is enough to solve a constraint satisfaction problem. In our example, if *x*_1 _= [2, 3]*, x*_2 _= [4, 6] and *x*_3 _= [0, 20], the propagators can only narrow the domain of *x*_3 _to [6, 9] by discarding the unfeasible values [0, 5] (resp. [9, 20]) in the lower (resp. upper) part of the interval. At this point, when no further propagation is possible, the search engine chooses a variable *x *= [*l, u*] such that *u − l *is greater than a given precision *E*. Then, the problem is split into two: one searching for a solution considering *x *= [*l, u/*2) and another considering *x *= [*u/*2*, u*] where *u/*2 is the mid-point. This is known in Constraint Programming as *labelling *or *enumeration *and leads to a depth-first search strategy.

The combination of constraint propagation and enumeration yields a complete solution method: all solutions generated are indeed solutions to the problem and, if a solution exists, the procedure will eventually find it. In our previous example, the domains (*intervals*) *x*_1 _= [3, 3], *x*_2 _= [6, 6], *x*_3 _= [9, 9] are one of the possible solutions for the constraint *x*_1 _+ *x*_2 _= *x*_3_.

**Figure 3:**
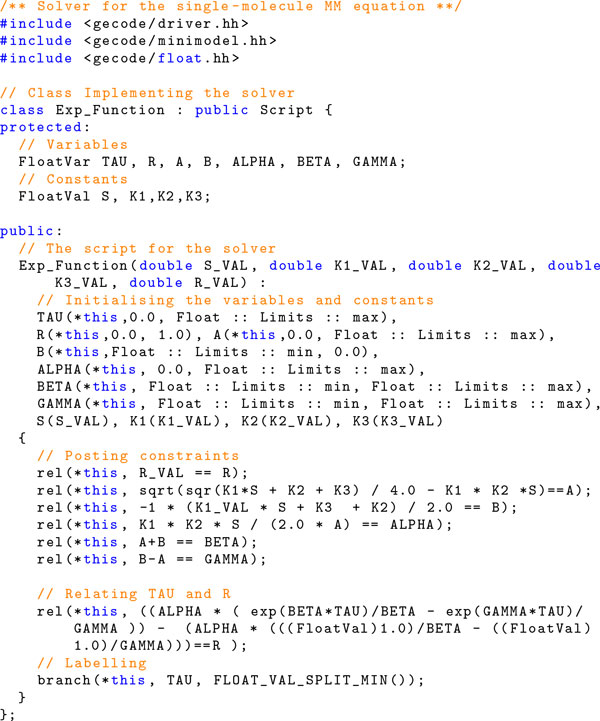
**Gecode script for solving *r *= *F *(*τ *) in the Equation (3)**.

Now we show how to find a solution for Equation (3) by using the Constraint Programming approach implemented in Gecode. The main parts of the needed code are in Figure 3 and explained below.

Lines 2-4 include the required libraries from Gecode. Line 7 defines the class Exp Function as a subclass of the the Gecode class Script. Hence, an instance of the class Exp Function can be used to solve our problem since this class actually defines the solver, i.e., the variables and the constraints needed.

The variables (of type FloatVar) are declared in line 10. The required constants, i.e., the parameters of the function, are declared in line 12. In lines 16-39 we declare the constructor of the class. The constructor takes as parameters the concentration (i.e., *S*) and the rates *k*_1_*, k*_2 _and *k*_3_. The first parameter *opt *is needed in Gecode to determine the number of solutions that the solver must generate. Lines 18-24 initialise the variables. Note for instance that TAU is a variable that can take any value greater than 0.0. As we know, B (see Equation (3)) cannot be positive and so this variable is initialised in the interval (*−∞*, 0.0]. The same holds also for the other variables.

In lines 27-35 we add the needed propagators to solve the equation *F *(*τ *) = *r*. For instance, in line 35 we establish the relation between R and the rest of variables. As we already explained, such a relation allows us to both determine *r *given a *τ *and compute *τ *given some *r *(the inverse of the function needed here). We note that we simply write the relations as the equations presented in the previous section by using the appropriate notation such as, for instance, sqrt to denote the square root and exp to denote the exponential function.

The labelling strategy is in line 37 where we specify that the variable TAU is chosen for labelling and the values not greater than the mid-point are explored first.

It is worth mentioning some advantages of using Constraint Programming instead of classical numerical methods to solve *F *(*τ *) = *r*. The most important one is that in Constraint Programming, interval arithmetic allows us to bound numeric errors appearing when doing calculations. Such accuracy is the result of representing a real number by means of an interval [*l, u*] instead of a single floating-point number. Hence, when a solution is found, we have a guarantee that the result is indeed a solution satisfying all the constraints. Another advantage is that in Constraint Programming, it is possible to find all the solutions for a given set of constraints. Moreover, due to the labelling process, the method is complete, i.e., if there is a solution, the solver eventually will find it. Finally, in constraint solving no initial parameters for iteration are required (see [22,23] for further details).

### Implementation

Now we are ready to show how to implement our technique to simulate biochemical reactions. The input of our tool is a biochemical system specified through a set of reactions having the generic:

(7) $$a_{\text{1}}X_{\text{1}}+\ldots +a_{n}X_{n}\overset{k}{\underset{}{\to }}b_{\text{1}}Y_{\text{1}}+\ldots +b_{m}Y_{m}$$

where the constants *a*_1_*, ..., a_n _*and *b*_1_*, ..., b_m _*are the stoichiometric coefficients. *k *stands for the kinetic rate constant. Therefore, *a*_1_*X*_1_*, ..., a_n_X_n _*are reactants that interact (and are consumed) yielding to the products *b*_1_*Y*_1_*, ..., b_m_Y_m_*. In addition, for each reaction we can define the corresponding PDF (with its needed parameters) that will be used to sample the duration of a particular reaction.

Besides the parser which loads the input, the tool is composed by two main modules that work together. One module is invoked each time we want to compute a solution for *F *(*τ *) = *r*. It takes as input the concentrations of the reactants, and a reaction; then it chooses the suitable PDF and it uses the Constraint Programming approach to compute the solution. The other module is the core engine of the tool:

it takes the value of *τ *for each reaction for which there are enough reactants in the system and then it chooses which reaction will take place, selecting the one with the smallest *τ *value. At this point, it simulates the designated reaction by consuming the reagents and adding to the system the products. After that, *τ *is added to the current time and it starts over again. In this way, given a set of reactions and a set of concentrations, we are able to simulate the evolution of the modelled system. The output of the tool is a list comprising a time stamp, the concentrations at that time, and the last reaction used.

## Results and discussion

To show how our method works we report on two experiments consisting of generating random samples of Equation (3) and comparing the sampled data with the PDF used.

**Example 1 (Performing the sampling) ***Consider the following set of parameters taken from *[17]:

$$\begin{array}{cccccc} A_{1}:k_{1} & =10^{7}M^{-1}s^{-1} & A_{2}:k_{1} & =10^{7}M^{-1}s^{-1} & A_{3}:k_{1} & =10^{7}M^{-1}s^{-1}\\ k_{2} & =250s^{-1} & k_{2} & =250s^{-1}\; & k_{2} & =250s^{-1}\\ k_{-1} & =0s^{-1} & k_{-1} & =50s^{-1} & k_{-1} & =2000s^{-1}\\ \;\left[S\right] & =0.005mM & \left[S\right] & =0.005mM & \left[S\right] & =0.005mM \end{array}$$

**Figure 4:**
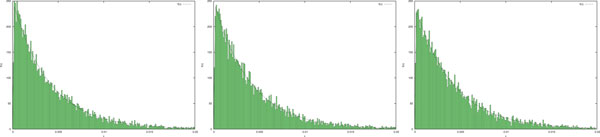
**Histograms representing the occurrences of *τ *obtained from the code in Figure 3 which provides samples of *τ *according to the density function in Equation (3)**. The continuous line represents the function in Equation (3) calculated using the parameters in Example 1. The graphs should be compared with the results obtained in [17].

*By using the code in *Figure 3*, we obtained 1000 samples of τ. The histogram of the resulting values and the plot of the Equation *(3) *for the three sets of parameters is depicted in *Figure 4.

The second example is used to illustrate how our method can be used for simulating reactions catalysed by single enzymes based on the parameters studied in [31]. That paper provides kinetic parameters referring to the enzyme *β*-lactamase which is present in bacteria such as *S. aureus *and *E. coli*. This enzyme hydrolyses benzylpenicillin so preserving the micro-organisms from being killed by this antibiotic. The kinetic parameters from [31] are not measured from single-molecule assays but rather from traditional "ensemble" observations, in which reactants and enzymes are allowed to react in concentrations of the order of moles per litre. Our aim is to verify whether our method, which satisfies the relation expressed in Equation (3), can be used to perform simulations also using data coming from ensemble experiments which are the most frequently present in the literature.

**Example 2 (Simulating single-molecule reactions) ***Let us consider the following set of parameters reported in *[31]:

$$\begin{array}{cccccc} B_{\text{1}}:k_{\text{1}} & =\text{41}\mu M^{-1}s^{-1} & B_{\text{2}}:k_{\text{1}} & =\text{22}\mu M^{-1}s^{-1} & B_{\text{3}}:k_{\text{1}} & =\text{123}\mu M^{-1}s^{-1}\\ k_{\text{2}} & =\text{192}0s^{-1} & k_{\text{2}} & =\text{62}s^{-1} & k_{\text{2}} & =\text{98}0s^{-1}\\ k_{-1} & =\text{232}0s^{-1} & k_{-1} & =\text{196}s^{-1} & k_{-1} & =\text{118}00s^{-1} \end{array}$$

**Figure 5:**
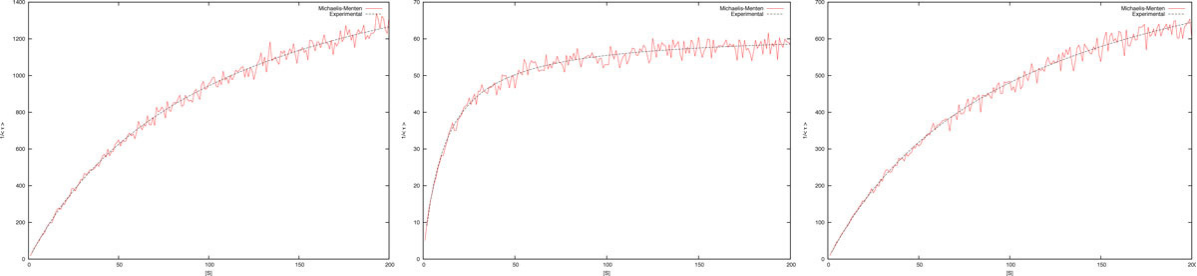
**Plots of [*S*] vs $\frac{1}{\left\langle \tau \right\rangle }$ using the parameters in Example 2**. The red (continuous) lines represent the MM equations calculated with the same (experimentally evaluated) parameters used for the probability density functions.

*We range *[*S*] *from *0.001 *to *200*. For each value of *[*S*] *we compute the average of 100 values for τ (denoted by$\langle \tau \rangle \;$ ). In *Figure 5*we show the plot of *[*S*] *vs $\frac{1}{\left\langle \tau \right\rangle }$along with the MM saturation curve obtained from Equation *(4).

As shown in Figure 5 and confirmed by the good results of a fit test (Pearson's *χ*2 test, with *p <*0.005), the values of *τ *we obtained through our method are consistent with the underlying MM model which is represented in each Figure together with the respective $\frac{1}{\left\langle \tau \right\rangle }$ plot. These results, again, show that the technique we propose allows us to faithfully simulate in one step the occurrence of an enzymatic reaction directly using the data coming from the literature.

The method we have presented for sampling probabilistic distribution functions can be generally applied to other ones such as Exponential (a.k.a. negative exponential distribution) and Erlang by using the same parameters reported in [14].

**Figure 6:**
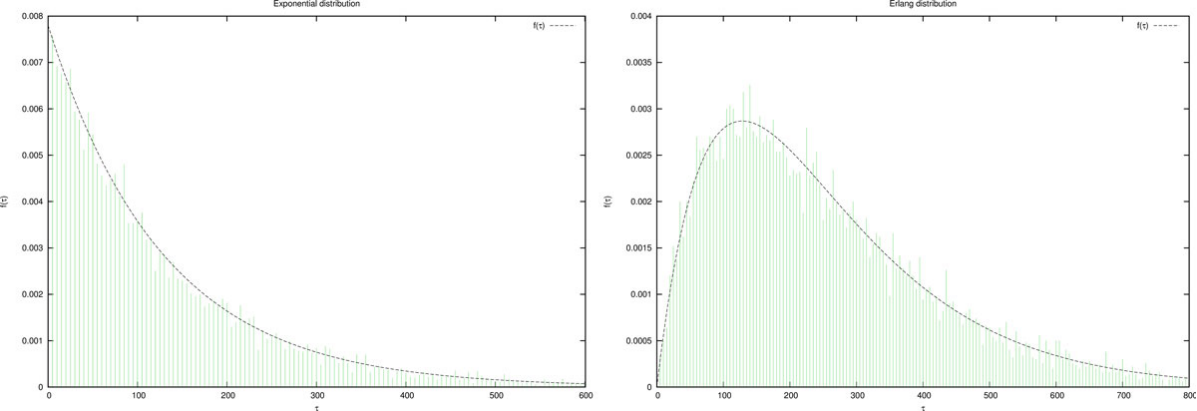
**Histograms represent the samples of *τ *according to the density functions and parameters in Examples 3 and 4**.

**Example 3 (Exponential PDF) ***Let f *(*τ *) = *λ e^−λτ ^for τ ≥ *0 *be an exponential PDF and F *(*τ *) = 1 *− e^−λτ ^be the corresponding cumulative distribution function*. Figure 6*(a) shows the histogram of the occurrence of τ obtained with a Gecode code similar to that in *Figure 3*. The parameter λ is set to *0.0078.

**Example 4 (Erlang PDF) ***Let f *(*τ *) *and F *(*τ *) *be an Erlang PDF (resp. cumulative PDF) defined as:*

$$f\left(\tau \right)=\frac{\lambda^{k}{x^{k-}}^{\text{1}}e^{-\lambda x}}{\left(k-\text{1}\right)!}\;\;F\left(\tau \right)=\text{1}-\overset{k-\text{1}}{\underset{n=0}{\sum }}\frac{1}{n!}e^{-\lambda x}{\left(\lambda x\right)}^{n}$$

*with the parameters k *= 2 *and λ *= 0.0078. Figure 6*(b) shows the histogram of the occurrence of τ obtained with a Gecode code similar to that in *Figure 3.

Now let us show how our method can be used to simulate a network of biochemical reactions.

**Example 5 (A simple Network) ***Consider the following elementary reactions:*

(1) *A → B *(2) *A → C*

**Figure 7:**
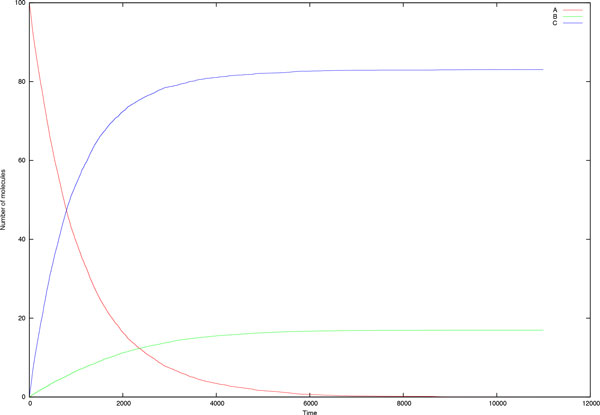
**Temporal trace of the species concentrations in a simple network of two reactions: (1) *A → B*, (2) *A → C***. PDFs and parameters are described in Example 5.

*Initial concentrations are A *= 100*, B *= 0 *and C *= 0*. Reaction (1) follows the PDF in Equation *(3) *with parameters k*_1 _= 1.0*, k*_2 _= 1.0 *and k*_*−*1 _= 10.0*. In reaction (2) τ is sampled from the exponential PDF f *(*τ *) = 10[*A*]*e−*[*A*]*τ. The results are depicted in *Figure 7.

Comparing Markovian and non-Markovian simulations

In this section we analyse the differences emerging in describing the reaction set in Equation (1) through two different DS models. In one case, we use an approach *àla *Gillespie, lumping the reaction scheme in Equation (1) into one *S → P *reaction. In this scenario, the occurrence of a reaction is seen as a single Markov transition with the waiting time distributed accordingly to a negative exponential PDF. The propensity *a *= *v *is calculated from Equation (2), as suggested in [13]. In the other case, we reduce the reaction scheme in Equation (1) to a single *S → P *reaction but the waiting times are computed (using our method) according to Equation (3), thus obtaining a non-Markovian model.

**Figure 8:**
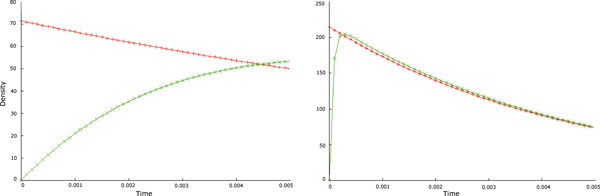
**Plots of the negative-exponential PDF (red) and the PDF according to Equation (3) (green)**. The plot on the left ([*S*] vs. *v*) is obtained with low *S*_0 _and with *k*_*−*1 _<<*k*_2 _while the plot on the right ([*S*] vs.$\frac{1}{\left\langle \tau \right\rangle }$) with higher *S*_0 _and *k*_2 _>*k*_*−*1_.

As previously noticed, experimental evidence provided by studies on single molecule experiments suggests that the occurrence of a reaction catalysed by single enzymes when observed at the mesoscopic level (i.e., as a single *S → P *reaction) exhibits non-Markovian dynamics. Our aim here is to evaluate the impact of the approximation introduced by simulating single-molecule enzymatic reactions as a Markov process through the GSSA. To do this, we first compare the trend of the two PDFs for waiting times (the negative exponential with propensity *a *and Equation (3)) when the initial values of *S *(*S*_0_), *k*_1_, *k*_*−*1 _and *k*_2 _are varied. It can be noticed in Figure 8 that the differences between the two PDFs mainly concern the low values of waiting times: according to the negative exponential PDF, waiting times close to zero have higher values of *F *(*τ *), while accordingly to the PDF in Equation (3) the higher values for *F *(*τ *) are not close to zero. Noticeably, these differences are greater when *S*_0 _is low and when *k*_*−*1 _is significantly less than *k*_2_. This implies that, with respect to the experimental findings on single-molecule enzymatic reactions, the Markovian approach introduces more approximations when the modelled system is composed of a low number of reactants and when the kinetic characteristics of the enzymatic reaction (i.e., various combinations of the values of *k*_1_, *k*_*−*1 _and *k*_2_) prevent the system from quickly reaching the steady state.

**Figure 9:**
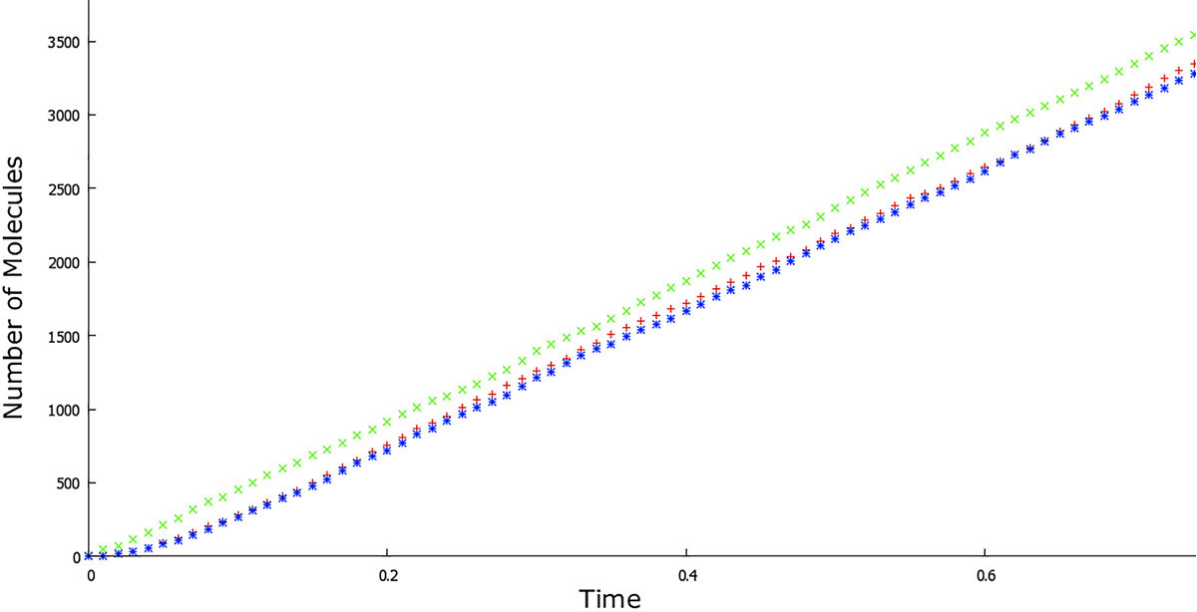
**Plots of number of product molecules vs**. time obtained simulating a single-molecule reaction through a Markovian (green) or non-Markovian (red) single jump. The blue plot is obtained describing the corresponding full model. The parameters were taken from [13].

To study the impact of the differences amongst PDFs on the dynamics of the system, we simulated the occurrence of single-enzyme catalysed reactions as single *S → P *Markov or non-Markov jumps. We compared the obtained results with the outputs of simulations performed describing the set of reactions in Equation (1) through the GSSA specifying each single step (call it *full model*). As proposed in [13], given the correctness of GSSA, the full model can be used as a convenient benchmark for evaluating the precision of simulations against the MM model. As can be noticed in Figure 9 (and confirmed by Pearson's test), the results of our simulations fit the full model significantly better than the corresponding Markovian approach. Thus, our technique allows us to describe the considered enzymatic reaction as a single *S → P *step with an accuracy comparable to that of the full model.

### Comparison with other systems

In this section, we compare the computational cost of our method with respect to Gillespie's Direct Method (DM). Our interest here is to consider only those implementations of GSSA which provide exact solutions as our method does. Thus, we will not consider approaches such as the tau-leaping [32] that can be computationally less costly but provides approximate solutions of the CME. The other exact implementation of GSSA, namely *first reaction method *(FRM), is known to be computationally more expensive than the DM and hence does not represent a good benchmark for our comparative tests. The same rationale applies also to Gibson and Bruck *next reaction method *which, in essence, is an optimisation of the FRM and has been shown to have a worse performance than the so-called Optimised Direct Method (ODM) [33].

We shall consider two different implementations of Gillespie's DM: (1) an implementation in the package R of GSSA as-it-is ( http://cran.r-project.org/ web/packages/GillespieSSA/) and (2) an implementation of the ODM provided by *Stochkit*, a popular extensible of the algorithm as-it-is, provided by the "GillespieSSA" package for the R suite ( http://cran.r-project.org/web/packages/ GillespieSSA/) and (2) an implementation of Gillespie's ODM provided by *StochKit*, a popular extensible stochastic simulation framework developed in C++ (http://stochkit.sourceforge.net). We choose Gillespie's ODM as representative of a group of methods, such as *logarithmic direct method *[34], *sorting direct method *[35] and a tailored version of a kinetic Monte-Carlo method [36], which aim at optimising Gillespie's DM through various heuristics that reduce the complexity of finding the next reaction to be fired. According to the literature, the computational performances of these methods are similar. Hence, we chose to consider only the ODM whose implementation in *StochKit *is well known and verified by the users.

Comparing our approach with both a native and an optimised version of the DM will provide more complete information. The ODM, in particular, increases the efficiency of the reaction-selection step in which the random number *r *is generated (i.e., index value), which is a key bottleneck in the DM. This is done in the ODM by pre-ordering the reactions so that those with larger propensity functions have smaller index values in the search list so that reactions occurring more frequently are higher up (hence applied first), thus reducing the search depth of the linear search. Moreover, the ODM only updates the propensity functions that change and a pre-simulation step is performed in order to ascertain the relative frequencies of each reaction.

In our comparative tests, we considered two different scenarios: (1) the MM reaction scheme; (2) a linear chain of reactions. All the simulations presented here were performed on an iMac 2.9GHz, with a quad-core Intel Core i5 and 8GB of RAM. The data reported correspond to the average of executing the tests 10 times.

#### Test set 1: the Michaelis-Menten reaction scheme

In the previous subsections we showed that, using our approach, we can safely lump the MM reaction scheme into one *S → P *reaction. We also showed that, for achieving the same accuracy exhibited by our method, the corresponding simulation *a la *Gillespie must be performed considering explicitly all the reactions of the MM scheme (call it *full Gillespie*). Given these results, for analysing the computational cost of our approach, we performed a series of tests simulating the occurrence of enzymatically catalysed reactions. We assumed that the considered enzyme exhibits MM kinetics and thus we compared our method versus the corresponding full Gillespie approach. Our aim here is to show that our method allows us to safely lump the MM reaction scheme into a single *S → P *reaction without significant loss of computational efficiency.

We found that our method outperforms the corresponding Gillespie approach when the reaction system becomes more stiff, i.e., the reaction rates will assume significantly different values spanning various orders of magnitude. To show this, we performed different tests, varying the value of the *k*_2 _parameter. As *k*_2 _takes lower values (keeping *k*_1 _and *k*_*−*1 _constant) the system becomes more stiff. For each value of *k*_2_, we recorded the computational time and the number of iterations needed for obtaining 500 molecules of the product *P *, starting from 10^6 ^molecules of *S *and 10^2 ^molecules of *E *. The results of these tests are shown in Table 1. Noticeably, our method outperforms the native R implementation for each one of the selected values of *k*_2_. Moreover, it turns out that when *k*_2 _becomes smaller our method outperforms also the *StochKit *implementation. This can be explained by the following facts. As the value of *k*_2 _decreases, the values of the sampled *τ *also decreases. Hence, the simulation time needed to reach a given amount of the product (in this case 500 units) is greater. In our approach, the number of iterations for producing the 500 units remains the same since in each case (independently of the parameters) we always need to sample the PDF 500 times. On the contrary, GSSA requires more iterations to deal with simulations where the simulated time is longer. Hence, the computational time grows.

**Table 1:** Comparisons between StochKit, the SSA implementation on the R-System and our approach for a MM reaction scheme.

	Gecode		R-System		StochKit
2	Time	Iterations	Time	Iterations	Time Iterations

10^0^	0.28	500	1.10	10412.40	0.04 5

10^*−*1^	0.35	500	28.78	99929.2	0.11 48

10^*−*2^	0.39	500	1855.92	990849.5	0.26 500

10^*−*3^	0.43	500	-	-	0.78 5000

10^*−*4^	0.42	500	-	-	7.75 50000

10^*−*5^	0.39	500	-	-	77.50 500000

Times in seconds reported correspond to the average of 10 runs. Constants *k*_1 _and *k*_3 _were set to 10 and *k*_2 _varies as shown in the table. The initial concentrations of *S *and *E *were set to 100000 and 100 molecules, respectively.

#### Test set 2: a chain of reactions

In this test we aimed to investigate the variations of the computational effort needed for performing simulations when the number of reactions composing the system grows. To do this, we tested both our method and the StochKit implementation of the ODM against simple systems composed of "chains" of (bio)chemical reactions, i.e., series of reactions in which the product of a given reaction becomes the reactant of the following. In each of our tests, the number of reactions composing the chain was increased by one. The smallest system comprised a single *A → B *reaction, while the largest system we considered comprised twenty reactions. In each simulation all the parameters, namely the rate constants and the initial amount of reactants were kept constant, allowing only the length of the "chain" to vary. The results are reported in Table 2. It turned out that our method scales better than StochKit when the number of reactions grows in the tests performed. Moving from *n *to *n *+ 1 reactions, in our case, implies that an extra value of *τ *for the new reaction must be computed. Moreover, the linear search for the lowest value of *τ *needed in each iteration must consider an array of *n *+ 1 positions. We note that StochKit is a more general tool than the one we are proposing here including, for instance, different simulation algorithms. Hence, it could be the case that more complex data structures are involved. This may explain why by increasing the number of reactions we get a better performance than the one exhibited by StochKit for the set of experiments reported in Table 2.

**Table 2:** Comparisons between StochKit and our approach for a chain of *N *reactions of the shape *A_i _→ A*_*i*+__1_.

**N**.	Gecode	StochKit
1	0.008	0.019

2	0.012	0.034

3	0.012	0.056

4	0.014	0.086

5	0.015	0.126

6	0.017	0.167

7	0.018	0.208

8	0.02	0.26

9	0.021	0.3

10	0.022	0.354

11	0.022	0.424

12	0.027	0.447

13	0.028	0.494

14	0.028	0.545

15	0.03	0.592

16	0.033	0.654

17	0.033	0.714

18	0.037	0.758

19	0.039	0.818

20	0.039	0.87

In each case, the kinetic constants were set to 1.0, the initial amount of each component is 100000 molecules and the simulation time corresponds to 5 time-units.

## Conclusions

In this paper, we presented a method for describing stochastic simulations of nonMarkovian processes. In particular, we used this method for sampling waiting times from general distributions and from a PDF inferred from *wet-lab *experiments regarding reactions catalysed by single-enzyme molecules. For this case-study, we provided a simulation algorithm which has some advantages over the corresponding Markovian approach. Specifically, it turned out to be more precise in those cases characterised by a low number of molecules and by a dynamics that makes the steady-state not quickly reachable. Encouraged by these findings, we are planning to use our technique for performing case-specific discrete-state continuoustime non-Markovian algorithms for sampling waiting times from experimentally inferred PDFs. Moreover, we are currently embedding the algorithm proposed here in BioWayS [37,38], our piece of software designed for modelling and simulating biochemical processes. Another interesting issue for future work would be to study through our method how competition for enzymes by different reactions may affect the overall dynamics of the investigated system.

## List of abbreviations

PDF: probability density function; DS: discrete stochastic; CTMP: continuous-time Markov process; CDM: continuous deterministic modelling; (G)SSA: (Gillespie) stochastic simulation algorithm; ITS: inverse transform sampling; CME: chemical master equation; RPDF: reaction probability density function; BWB: beta workbench; MM: Michaelis-Menten; ODEs: ordinary differential equations; ODM: optimised direct mehod; DM: direct mehod.

## Competing interests

The authors declare that they have no competing interests.

## Authors' contributions

DC, MF, DH and CO designed the framework. DH and DC selected the case studies, and discussed them with CO, MF and LT. CO and LT designed and implemented the software tools, discussing them with DC, MF and DH. All authors contributed to write the manuscript. All authors read and approved the final manuscript.

## Declarations

Publication of the article was funded by Moreno Falaschi and Davide Chiarugi.

This article has been published as part of *BMC Systems Biology *Volume 9 Supplement 3, 2015: Proceedings of the Italian Society of Bioinformatics (BITS): Annual Meeting 2014: Systems Biology. The full contents of the supplement are available online at http://www.biomedcentral.com/bmcsystbiol/supplements/9/S3.
